# Internalization of Heterologous Sugar Transporters by Endogenous α-Arrestins in the Yeast Saccharomyces cerevisiae

**DOI:** 10.1128/AEM.02148-16

**Published:** 2016-11-21

**Authors:** Arpita Sen, Ligia Acosta-Sampson, Christopher G. Alvaro, Jonathan S. Ahn, Jamie H. D. Cate, Jeremy Thorner

**Affiliations:** a Department of Molecular and Cell Biology, University of California, Berkeley, California, USA; b Department of Chemistry, University of California, Berkeley, California, USA; c Energy Biosciences Institute, University of California, Berkeley, California, USA; d School of Public Health, University of California, Berkeley, California, USA; University of Buenos Aires

## Abstract

When expressed in Saccharomyces cerevisiae using either of two constitutive yeast promoters (*PGK1_prom_* and *CCW12_prom_*), the transporters CDT-1 and CDT-2 from the filamentous fungus Neurospora crassa are able to catalyze, respectively, active transport and facilitated diffusion of cellobiose (and, for CDT-2, also xylan and its derivatives). In S. cerevisiae, endogenous permeases are removed from the plasma membrane by clathrin-mediated endocytosis and are marked for internalization through ubiquitinylation catalyzed by Rsp5, a HECT class ubiquitin:protein ligase (E3). Recruitment of Rsp5 to specific targets is mediated by a 14-member family of endocytic adaptor proteins, termed α-arrestins. Here we demonstrate that CDT-1 and CDT-2 are subject to α-arrestin-mediated endocytosis, that four α-arrestins (Rod1, Rog3, Aly1, and Aly2) are primarily responsible for this internalization, that the presence of the transport substrate promotes transporter endocytosis, and that, at least for CDT-2, residues located in its C-terminal cytosolic domain are necessary for its efficient endocytosis. Both α-arrestin-deficient cells expressing CDT-2 and otherwise wild-type cells expressing CDT-2 mutants unresponsive to α-arrestin-driven internalization exhibit an increased level of plasma membrane-localized transporter compared to that of wild-type cells, and they grow, utilize the transport substrate, and generate ethanol anaerobically better than control cells.

**IMPORTANCE** Ethanolic fermentation of the breakdown products of plant biomass by budding yeast Saccharomyces cerevisiae remains an attractive biofuel source. To achieve this end, genes for heterologous sugar transporters and the requisite enzyme(s) for subsequent metabolism have been successfully expressed in this yeast. For one of the heterologous transporters examined in this study, we found that the amount of this protein residing in the plasma membrane was the rate-limiting factor for utilization of the cognate carbon source (cellobiose) and its conversion to ethanol.

## INTRODUCTION

Ethanol is a widely used, environmentally clean, and renewable biofuel produced by microbial fermentation of sugar sources derived from food-related crop plants, such as corn and sugar cane, referred to as “first-generation” ethanol (1, 2). An alternative source of ethanol that avoids the “food versus fuel” ethical conflict is sugar derived from non-crop plant biomass, referred to as “second-generation” ethanol (2, 3). Plant biomass is composed of lignocellulosic material, which consists of cellulose (the most abundant fraction), hemicellulose, and lignin (4). For its fermentation to occur, lignocellulosic biomass is first pretreated to make its components more accessible to breakdown and then hydrolyzed either enzymatically or chemically to release fermentable sugars (5). The principal sugars liberated by hydrolysis of cellulose consist of cellodextrins and glucose, whereas hydrolysis of hemicelluloses releases primarily xylans and xylose.

To produce ethanol as a biofuel, industrial strains of budding yeast Saccharomyces cerevisiae are primarily used (6, 7). Native S. cerevisiae, although unable to efficiently utilize xylose (8, 9), is proficient in the utilization and fermentation of glucose. However, large-scale enzymatic degradation of cellulose into glucose is expensive, requiring, first, hydrolysis of cellulose by cellulases to generate the β(1→4)-linked disaccharide cellobiose (and higher cellodextrins) and then subsequent cleavage of cellobiose into glucose by β-glucosidases. Aside from the expense, complete enzymatic conversion of cellulose to glucose is problematic because high glucose concentrations inhibit both cellulases and β-glucosidases (10, 11). One approach that reduces cost, eliminates glucose-mediated inhibition of enzymes, and facilitates cofermentation of nonglucose sugars is based on the successful uptake of cellobiose, which is subsequently broken down to glucose after its transport into the cell. This end was achieved by ectopic coexpression in yeast of the gene for a cellobiose/cellodextrin transporter, either CDT-1 (NCU00801) or CDT-2 (NCU08114), and the gene for an intracellular β-glucosidase (*gh1-1*, NCU00130) from the filamentous fungus Neurospora crassa (12). CDT-1 catalyzes active transport of cellobiose, and CDT-2 mediates entry of cellobiose (as well as xylans) by facilitated diffusion (12, 13). Cellobiose fermentation, like fermentation of other nonglucose sugars in S. cerevisiae, occurs at a substantially lower rate than glucose fermentation under the same conditions, although cellobiose fermentation eventually reaches a yield of ethanol similar to that of glucose fermentation (14–17). Nonetheless, the lower rate of cellobiose fermentation hampers the application of this strategy at an industrial scale. Earlier work has shown, though, that the efficiency of cellobiose utilization can be significantly improved by driving the increased expression of CDT-1 and GH1-1 using strong constitutive promoters (17).

In S. cerevisiae, endogenous nutrient permeases in the plasma membrane (PM), including sugar transporters, are marked for endocytosis through ubiquitinylation by the ubiquitin:protein ligase (E3) Rsp5 (18–20). Rsp5 associates with the PM via its N-terminal phospholipid-binding C2 domain and binds to potential targets via three internal WW domains that recognize the motif PPXY (and variants thereof, such as VPXY) (21, 22). However, many cargo proteins that undergo Rsp5-dependent ubiquitinylation do not contain the PPXY consensus (19). Indeed, endocytosis of most integral PM proteins is brought about through recruitment of Rsp5 via a family of endocytic adaptor proteins called α-arrestins (also referred to as arrestin-related trafficking adaptors or ARTs) (23, 24). In S. cerevisiae, this family comprises 14 members, which are characterized by an N-terminal cargo-binding arrestin fold domain and a C-terminal Rsp5-binding extension containing multiple PPXY motifs (23–25). The products of several members of the *HXT* family of hexose transporter genes in S. cerevisiae are endocytosed upon exposure to and transport of extracellular glucose (or glucose analogs) via their interaction with specific α-arrestins and subsequent ubiquitinylation by Rsp5 (20, 25, 26). N. crassa CDT-1 and CDT-2 belong to the same transporter family as the *HXT* transporters, namely, the sugar porter (SP) subfamily (Transporter Classification Database identifier 2.A.1.1; http://www.tcdb.org) of major facilitator transporters (27). Thus, it seemed plausible that α-arrestin-mediated downregulation of CDT-1 and/or CDT-2 might remove them from the cell surface, thereby imposing a limitation on the efficacy of cellobiose utilization and ethanol production from this carbon source.

Hence, in this study, we explored whether CDT-1 and CDT-2 are subject to internalization mediated via the endogenous α-arrestins in S. cerevisiae. If so, we sought to determine which of the 14 α-arrestins is responsible for the downregulation, what other factors may influence this process, how ubiquitinylation may be involved, and whether the rate of transporter removal from the cell surface is sufficiently rapid to negatively affect the efficiency of cellobiose utilization and the generation of ethanol from it.

## MATERIALS AND METHODS

### Strains and plasmids.

Yeast strains (Table 1) and plasmids (Table 2) were constructed using standard genetic methods (28, 29). DNA amplification by PCR employed Phusion DNA polymerase (New England BioLabs, Ipswich, MA), and all constructs were verified by sequencing. *CDT-1* and *CDT*-2 were PCR amplified from cDNA synthesized from mRNA isolated from N. crassa (FGSC 2489) grown on minimal medium plus Avicel (microcrystalline cellulose) as the sole carbon source (12). The N. crassa cdt-1 and *cdt-2* genes were cloned into the pRS316 plasmid (*CEN URA3*) using the In-Fusion HD cloning kit (Clontech Laboratories, Inc., Mountain View, CA). These transporters were expressed under the control of the S. cerevisiae *PGK1* promoter (*PGK1_prom_*) and the *CYC1* terminator; all transporters were tagged with enhanced green fluorescent protein (eGFP) at the C terminus. For construction of the CDT-2^KR^ mutants, double-stranded gene fragments spanning regions encompassing each set of mutations were synthesized as gBlocks by Integrated DNA Technologies (Coralville, IA). These gene fragments (Table 3) were cloned into the *CDT-2* coding sequence in a linearized pRS316 plasmid under the control of the S. cerevisiae *CCW12_prom_* and a *CYC1* terminator by using the InFusion HD cloning kit (Clontech Laboratories, Inc., Mountain View, CA). The codon-optimized version of *GH1-1* was expressed in pRS315 plasmid (*CEN LEU2*) under the control of the *CCW12_prom_* and the *CYC1* terminator. Codon optimization of this gene has been described elsewhere (17).

**TABLE 1:** Yeast strains used in this study

Strain	Description	Reference or source
BY4741	*MAT***a** *leu2Δ0 ura3Δ0 his3Δ1 met15Δ0*	Yeast deletion collection (Open Biosystems, Inc.)
*9arr*Δ (EN60) derivative	*MAT***a** *ecm21*Δ::*KANMX csr2*Δ::*KANMX bsd2Δ rod1Δ rog3*Δ::*NATMX ygr068cΔ ldb19Δ aly1Δ aly2Δ ylr392c*Δ::*HIS3 his3 ura3Δ0 leu2Δ0*	24
*4arr*Δ derivative	*MAT***a** *leu2Δ0 ura3Δ0 his3Δ1 met15Δ0 aly1*Δ::*KANMX aly2*Δ::*KANMX rod1*Δ::*HYG rog3*Δ::*NATMX*	This study
*rim8Δ art5*Δ mutant	*MAT***a** *leu2Δ0 ura3Δ0 his3Δ1 met15Δ0 rim8*Δ::*KANMX art5*Δ::*KANMX*	22
*rod1Δ rog3*Δ mutant	*MAT***a** *leu2Δ0 ura3Δ0 his3Δ1 met15Δ0 rod1*Δ::*KANMX rog3*Δ::*KANMX*	22
*rod1Δ rog3Δ ldb19*Δ mutant	*MAT***a** *leu2Δ0 ura3Δ0 his3Δ1 met15Δ0 rod1*Δ::*KANMX rog3*Δ::*KANMX ldb19*Δ::*NATMX*	22
*ecm21Δ csr2*Δ mutant	*MAT***a** *leu2Δ0 ura3Δ0 his3Δ1 met15Δ0 ecm21*Δ::*KANMX csr2*Δ::*KANMX*	22
*aly1Δ aly2*Δ mutant	*MAT***a** *leu2Δ0 ura3Δ0 his3Δ1 met15Δ0 aly1*Δ::*KANMX aly2*Δ::*KANMX*	22
*art10*Δ mutant	*MAT***a** *leu2Δ0 ura3Δ0 his3Δ1 met15Δ0 art10*Δ::*KANMX*	Yeast deletion collection (Open Biosystems, Inc.)
*ecm21Δ csr2Δ rod1Δ rog3*Δ mutant	*MAT***a** *leu2Δ0 ura3Δ0 his3Δ1 met15Δ0 ecm21*Δ::*KANMX csr2*Δ::*KANMX rod1*Δ::*HYG rog3*Δ::*NATMX*	This study
*ecm21Δ csr2Δ aly1Δ aly2*Δ mutant	*MAT***a** *leu2Δ0 ura3Δ0 his3Δ1 met15Δ0 ecm21*Δ::*KANMX csr2*Δ::*KANMX aly1*Δ::*HYG aly2*Δ::*NATMX*	This study

**TABLE 2:** Plasmids used in this study

Plasmid	Description	Reference
CDT-1–GFP	*PGK1_prom_* CDT-1–GFP–His_6_ *CEN URA3*	This study
CDT-2–GFP	*PGK1_prom_* CDT-2–GFP–His_6_ *CEN URA3*	This study
GH1-1	*CCW12_prom_* GH1-1 *CEN LEU2*	This study
CDT-2 WT–GFP	*CCW12_prom_* CDT-2–GFP–His_6_ *CEN URA3*	This study
CDT-2 Nt^KR^–GFP	*CCW12_prom_* CDT-2(K6R K7R K31R K32R K38R)–GFP–His_6_ *CEN URA3*	This study
CDT-2 Mid^KR^–GFP	*CCW12_prom_* CDT-2(K231R K233R K243R K263R K274R K283R)–GFP–His_6_ *CEN URA3*	This study
CDT-2 Ct^KR^–GFP	*CCW12_prom_* CDT-2 Ct(K484R K493R K512R K522R)–GFP–His_6_ *CEN URA3*	This study
CDT-2^Trunc^–GFP	*CCW12_prom_* CDT-2^Trunc^–GFP–His_6_ *CEN URA3*	This study (see Fig. S5)

**TABLE 3:** Sense-strand sequence of the DNA fragments used to construct the indicated K-to-R mutants of CDT-2

Mutant	Description (amino acid changes)	DNA
CDT-2 Nt^KR^	CDT-2 (K6R K7R K31R K32R K38R)	5′-ATGGGCATCTTCAACAGGCGTCCCGTGGCTCAGGCCGTCGACCTCAATCAGATACAGGAGGAGGCTCCTCAGTTTGAGAGGGTTGACTGGAGAAGGGACCCCGGTCTTCGCAGACTCTACTTCTACGCCTTC
CDT-2 Mid^KR^	CDT-2 (K231R K233R K243R K263R K274R K283R)	5′-CCCCGTTTCCTCATCGCCAGAGACAGGCACGACGAGGCCCTCCACATCCTCGCCAGATACCACGCCAACGGCGACCCCAACCACCCCACCGTCCAGTTTGAGTTCCGCGAGATCCGTGAGACCATCCGCCTCGAGATGGAATCGACCCGTAACAGCAGCTACCTCGACTTCTTCAGAAGCCGCGGCAACCGCTAC
CDT-2 Ct^KR^	CDT-2 (K484R K493R K512R K522R)	5′-TTCATGTACGTCGAGACCAGAGGCCCCACGCTCGAGGAGCTTGCCAGAGTCATTGATGGCGATGAGGCCGATGTTGCCCACATCGACATTCACCAGGTCGAGAGAGAGGTGGAGATTCACGAGCATGAGGGCAGGTCTGTTGCTGAATTCGAT

### Growth conditions.

Strains were grown at 30°C in either rich (yeast extract-peptone [YP]) or synthetic (S) medium (30) containing 2% cellobiose (unless otherwise specified) with appropriate nutrient supplements to support growth and with certain nutrients omitted to maintain selection for plasmids. For the anaerobic growth assays and fermentation experiments, we used optimized minimal medium (oMM) lacking appropriate nutrients for plasmid selection (17); oMM contained 10 g/liter (NH4)_2_SO_4_, 1 g/liter MgSO_4_·7H_2_O, 6 g/liter KH_2_PO_4_, 100 mg/liter adenine hemisulfate, 1.7 g/liter yeast nitrogen base (YNB; Sigma-Aldrich, St. Louis, MO), 2× recommended CSM−Ura−Leu (complete supplement mixture lacking Ura and Leu) dropout mix (MP Biomedicals, Santa Ana, CA), 10 mg/liter inositol, 100 mg/liter glutamic acid, 20 mg/liter lysine, 375 mg/liter serine, 100 mM morpholineethanesulfonic acid (MES), pH 6. Glucose or cellobiose was added to this stock recipe depending on the experiment. Cellobiose and xylan from beechwood were obtained from Sigma-Aldrich (St. Louis, MO). Due to the difficulty of forming >5% stock solution of xylan, it was dissolved directly in YP (2%, wt/vol) with constant heating.

### Fluorescence microscopy.

Images were acquired using an Olympus BH2 microscope equipped with a charge-coupled-device (CCD) camera. For live imaging of cells expressing fluorescently tagged proteins (CDT-1–GFP and CDT-2–GFP), cell cultures were grown overnight at 30°C in synthetic (S) medium (30) containing the indicated carbon source (2% final concentration) with appropriate nutrient supplements to support growth and with certain nutrients omitted to maintain selection for plasmids. The following morning, cultures were diluted into a fresh sample of the same medium and grown to mid-exponential phase. Prior to imaging, cells were collected by a brief centrifugation and resuspended in a fresh sample of the same medium to form a dense suspension. Samples (8 μl) of this suspension were spotted onto a glass slide, covered with a coverslip (22 by 22 mm), and imaged with appropriate filters using identical imaging parameters. Image processing was performed with ImageJ software. For imaging under anaerobic conditions, cultures were grown as previously described and transferred to serum flasks, which were sealed with a rubber stopper and aluminum seal and purged under a constant stream of N_2_ gas for 30 min. These flasks were incubated with constant shaking at 30°C for 6 h. Slides were prepared, as described above, in an anaerobic chamber, after which the edges of the coverslips were carefully sealed with nail polish. Immediately after the seals dried, the slides were removed from the anaerobic hood and examined under the fluorescence microscope.

### Anaerobic growth assays.

All growth assays were performed using the Tecan Sunrise plate reader housed in an anaerobic chamber (Tecan, San Jose, CA) using biological triplicates. Single colonies of S. cerevisiae strains cotransformed with a pRS316 plasmid containing the transporter of interest and the pRS315 plasmid containing *GH1-1* were grown in oMM−Ura−Leu+Glc (Glc-supplemented oMM lacking Ura and Leu) to late exponential phase at 30°C in 24-well plates. Cultures were collected by centrifugation at 2,500 × *g*, and the supernatant fraction of the spent medium was discarded and the cell pellet resuspended in H_2_O. The measured *A*_600_ of the suspension was used to calculate the volume of oMM−Ura−Leu plus 8% (wt/vol) cellobiose to be inoculated to achieve an initial *A*_600_ of ∼0.1 for cultures (150-μl final volume) in clear, flat-bottom, 96-well plates. The plates were then sealed with a Microseal C optical seal (Bio-Rad, Hercules, CA), and the *A*_600_ was measured every 15 min for 96 h at 30°C with continuous shaking under these anoxic conditions.

### Fermentation assays.

Assessment of ethanol production by fermentation of oMM−Ura−Leu plus 8% cellobiose was performed in sealed serum flasks under anaerobic conditions. Yeast cells were cotransformed with plasmids containing the specified cellodextrin transporter and GH1-1 β-glucosidase. Seed cultures (10 ml) were grown in oMM−Ura−Leu+Glc overnight at 30°C and used to inoculate 200 ml of the same medium. After the cultures reached an *A*_600_ of 3 to 4, the cells were harvested by centrifugation at 4,000 × *g* for 10 min, washed with ultrapure H_2_O, pelleted at 4,000 × *g*, and resuspended in 40 to 50 ml of oMM−Ura−Leu plus 8% cellobiose. The final volume of oMM medium was adjusted so that the final culture density reached an *A*_600 nm_ of 20. The flasks were then sealed with a rubber stopper and aluminum seal and purged under a constant stream of N_2_ gas for 30 min. These flasks were incubated with constant shaking at 30°C. Sampling was performed using a sterile syringe inserted through the rubber stopper. Cellobiose and ethanol levels in the medium were measured by high-performance liquid chromatography in a Shimadzu chromatograph equipped with a refractive index detector and an RFP-fast acid column (100-mm length by 7.8-mm internal diameter; Phenomenex Inc., Torrance, CA, USA). The column was run in isocratic mode with 0.01 N H_2_SO_4_ mobile phase at a flow rate of 1 ml/min at 55°C.

## RESULTS

### Ectopically expressed cellobiose transporters CDT-1 and CDT-2 are internalized in an α-arrestin-dependent manner.

The related cellobiose transporters CDT-1 and CDT-2 (sequence alignment is shown in Fig. S1 in the supplemental material) have been successfully expressed in S. cerevisiae and, in concert with expression of the intracellular β-glucosidase GH1-1, allow this yeast to utilize cellobiose and ferment it (and higher cellodextrins) to ethanol (12). However, the steady-state level of any transporter in the PM is a balance between the rate of its synthesis and insertion and the rate of its endocytic removal. Hence, we reasoned that if either CDT-1 and/or CDT-2 is subject to rapid endocytosis in this heterologous host, this process might reduce the amount of the transporter available for mediating cellobiose uptake and thus might contribute to limiting the rate of cellobiose fermentation to ethanol.

**FIG 1:**
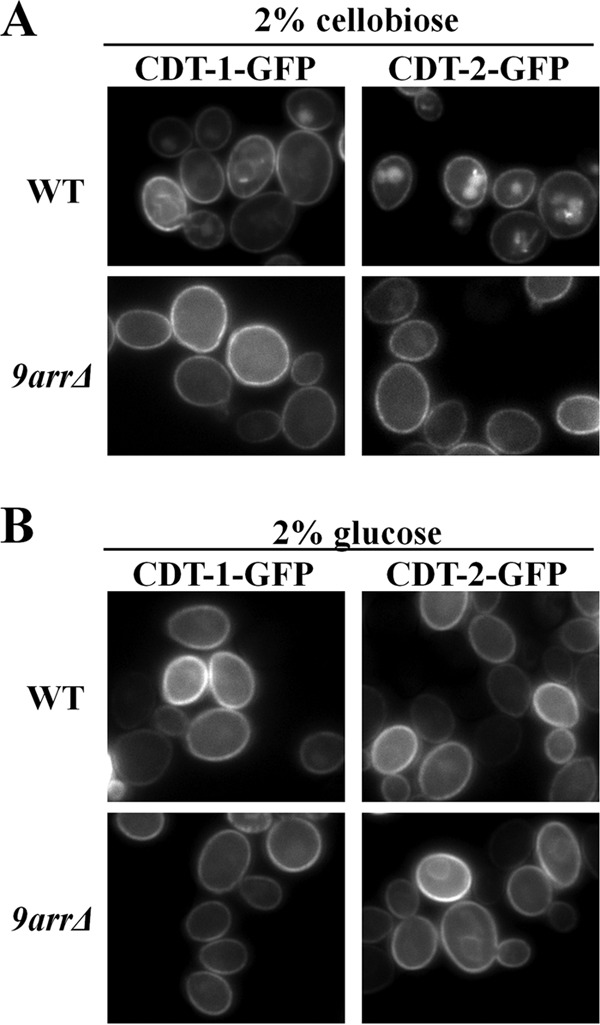
Cellobiose stimulates α-arrestin-dependent internalization of CDT-1 and CDT-2. Cultures of BY4741 (WT) and an otherwise isogenic strain harboring simultaneous deletions of 9 α-arrestin genes (termed *9arr*Δ cells) expressing either CDT-1–GFP or CDT-2–GFP were grown, and representative images were obtained in the presence of 2% cellobiose (A) or 2% glucose (B) as described in Materials and Methods. The cells coexpress the intracellular β-glucosidase (GH1-1).

To test whether either CDT-1 or CDT-2 is susceptible to α-arrestin-mediated Rsp5-dependent ubiquitinylation and subsequent internalization, we first examined whether the subcellular distribution of either transporter was influenced by the absence of α-arrestins. The final destination for internalized PM proteins is the vacuole, the yeast equivalent of the lysosome (31). For this purpose, we expressed CDT-1–eGFP and CDT-2–eGFP in control cells (strain BY4741; wild-type [WT] cells) coexpressing GH1-1 or in an otherwise isogenic BY4741 derivative expressing the same proteins but lacking 9 of the 14 α-arrestin genes (25), here called the *9arr*Δ strain. When grown on 2% cellobiose as the carbon source, we observed that in WT cells, CDT-1 and CDT-2 localized to both the PM and the lumen of the vacuole (Fig. 1A, upper panels), whereas in the *9arr*Δ strain, the transporters resided primarily at the cell surface with a marked reduction in the amount of these proteins in the vacuole (Fig. 1A, lower panels).

First, to quantify these observations, we scored a total of 200 cells in random fields of each culture for two features of every cell: its level of PM fluorescence and its level of vacuolar fluorescence. In agreement with the representative images shown in Fig. 1A, we found that a significant fraction (31%) of WT cells expressing CDT-1–GFP exhibited readily detectable vacuole-associated fluorescence (and concomitantly reduced PM fluorescence), whereas in the *9arr*Δ strain, the majority of the cells (96%) displayed very bright PM fluorescence and no discernible vacuolar fluorescence. Similarly, the majority (80%) of WT cells expressing CDT-2–GFP exhibited very robust vacuolar fluorescence (and concomitantly reduced PM fluorescence), and conversely, in the *9arr*Δ strain, 86% of the cells displayed bright PM fluorescence and very little or no detectable fluorescence in the vacuole. Second, we confirmed that the internal GFP fluorescence indeed represented a signal within the lumen of the vacuole by costaining with 7-amino-4-chloromethyl-coumarin (CMAC), a known marker for this compartment (20) (see Fig. S2 in the supplemental material).

Somewhat unexpectedly, we found that CDT-1 and CDT-2 internalization was promoted by the presence of their transport substrate, cellobiose, because when glucose was the carbon source, both transporters were PM localized and little vacuole-associated fluorescence was detected in either WT cells or the *9arr*Δ strain (Fig. 1B). Out of 200 total cells in random fields of each culture, we found that only 2% of WT cells expressing CDT-1–GFP in glucose medium exhibited robust vacuolar fluorescence and the majority (98%) exhibited very bright PM fluorescence and that, in the *9arr*Δ strain, all of the cells (100%) displayed only very weak vacuolar fluorescence and very bright PM fluorescence. Likewise, only 3% of WT cells expressing CDT-2–GFP in glucose medium exhibited readily detectable robust vacuolar fluorescence and the majority (97%) exhibited very bright PM fluorescence and, in the *9arr*Δ strain, only a very small fraction (1%) displayed readily detectable vacuolar fluorescence and the majority (99%) exhibited very bright PM fluorescence.

As has been observed for other heterologous integral membrane proteins expressed in yeast (32, 33), and indicative of some degree of misfolding or other kinetic delay in the trafficking of these transporters through the secretory pathway, we noted for both CDT-1–GFP and CDT-2–GFP a faint perinuclear fluorescent signal, which is the hallmark of some degree of accumulation of unfolded protein-chaperone complexes in the lumen of the endoplasmic reticulum (ER) (34, 35).

### Internalization of CDT-1 and CDT-2 requires joint action of the α-arrestins Rod1, Rog3, Aly1, and Aly2.

To determine whether the observed cellobiose-dependent endocytosis of CDT-1 or CDT-2 depends on any specific α-arrestin(s), we examined CDT-1 and CDT-2 localization in strains in which the genes for either a single α-arrestin or for paralogous pairs of α-arrestins were deleted. Among the 14 known S. cerevisiae α-arrestins, the following eight are apparent paralogs on the basis of sequence relatedness and overlapping function: Aly1/Art6 and Aly2/Art3, Csr2/Art8 and Ecm21/Art2, Art5 and Rim8/Art9, and Rod1/Art4 and Rog3/Art7 (23, 25). Hence, we examined CDT-1–eGFP and CDT2–eGFP localization in *aly1Δ aly2*Δ, *csr2Δ ecm21*Δ, *rim8Δ art5*Δ, and *rod1Δ rog3*Δ double mutant cells. We also examined the localization of these transporters in single mutant cells lacking either the α-arrestin Ldb19 or the α-arrestin Art10. However, we eliminated from our analysis the Art10 paralog Spo23, because it is expressed only in meiotic cells (36). Likewise, we did not examine the most distantly related α-arrestin-like proteins, Bul1 and Bul2, which have been implicated mainly in intracellular trafficking and sorting of permeases, such as the general amino acid permease Gap1 (37–39), as well as in their removal from the cell surface (40, 41).

**FIG 2:**
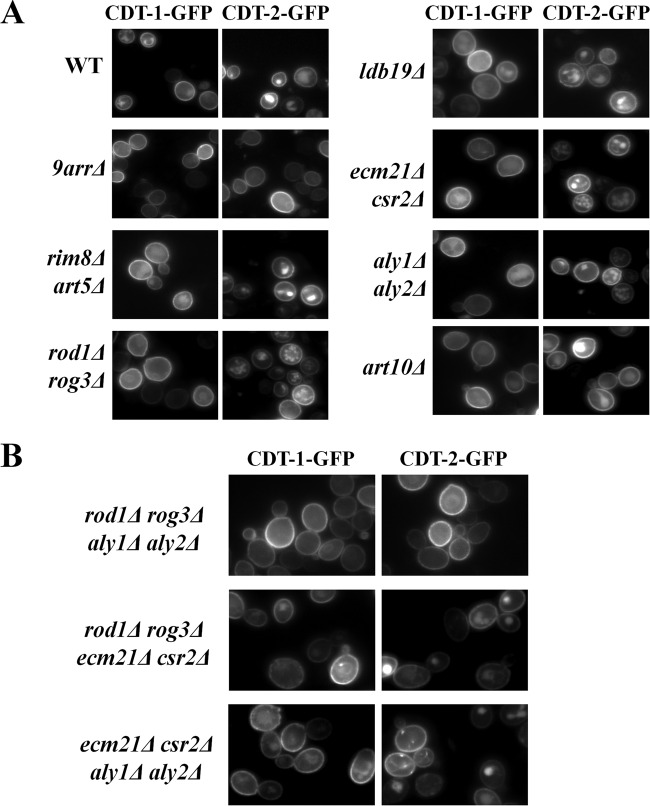
The α-arrestins Rod1, Rog3, Aly1, and Aly2 cooperate to drive the endocytosis of CDT-1 and CDT-2. (A) Cultures of BY4741 (WT), the *9arr*Δ strain, and otherwise isogenic strains with paralogous pairs (or more) of α-arrestin genes deleted were grown and imaged in the presence of 2% cellobiose as described in Materials and Methods. Each strain was cotransformed with the intracellular β-glucosidase (GH1-1) along with either CDT-1–GFP or CDT-2–GFP. (B) The same experiment as that described for panel A was performed, except the cells harbor quadruple deletions of α-arrestin genes.

We reasoned that if a specific α-arrestin(s) is important for CDT-1 and CDT-2 internalization, then in the absence of that α-arrestin(s), CDT-1 and CDT-2 should phenocopy the enhanced cell membrane localization and reduced vacuolar localization observed in the *9arr*Δ strain. However, in all of the α-arrestin-deficient strains tested, CDT-1 and CDT-2 localization was observed in the vacuole as well as at the cell membrane (Fig. 2A), suggesting that multiple sets of α-arrestins contribute to mediating the internalization of these two cellobiose transporters.

In this regard, we did note a modest decrease in vacuolar localization in both the *rod1Δ rog3*Δ cells and the *aly1Δ aly2*Δ cells. For 200 total cells in random fields of each culture expressing CDT-1–GFP, we observed the following percentages of cells with a robust vacuolar signal: *9arr*Δ cells, 4%; WT, 31%; *art10*Δ cells, 32%; *ldb19*Δ cells, 37%; *csr2Δ ecm21*Δ cells, 31%; *art5Δ rim8*Δ cells, 38%; *aly1Δ aly2*Δ cells, 26%; and *rod1Δ rog3*Δ cells, 29%. Similarly, for 200 total cells in random fields of each culture expressing CDT-2–GFP, we observed the following percentages of cells with a robust vacuolar signal: *9arr*Δ cells, 14%; WT, 80%; *art10*Δ cells, 82%; *ldb19*Δ cells, 82%; *csr2Δ ecm21*Δ cells, 83%; *art5Δ rim8*Δ cells, 84%; *aly1Δ aly2*Δ cells, 78%; and *rod1Δ rog3*Δ cells, 74%.

For this reason, we constructed an *aly1Δ aly2Δ rod1Δ rog3*Δ quadruple mutant of BY4741 (here called the *4arr*Δ strain). Reassuringly, localization of CDT-1 and CDT-2 in the *4arr*Δ strain displayed prominent PM fluorescence and markedly reduced vacuolar fluorescence, phenocopying the localization observed in the *9arr*Δ strain (Fig. 2B). Moreover, this result was not simply the cumulative effect of deleting the genes for a total of four α-arrestins, because the same effect was not observed when either *aly1Δ aly2*Δ or *rod1Δ rog3*Δ were combined with deletions of other pairs of paralogous α-arrestin genes (Fig. 2B). For 200 total cells in random fields of each culture expressing CDT-1–GFP, we observed the following percentages of cells with a robust vacuolar signal: *aly1Δ aly2 rod1Δ rog3*Δ cells, 7%; *rod1Δ rog3Δ csr2Δ ecm21*Δ cells, 37%; and *aly1Δ aly2 csr2Δ ecm21*Δ cells, 35%. Similarly, for 200 total cells in random fields of each culture expressing CDT-2–GFP, we observed the following percentages of cells with a robust vacuolar signal: *aly1Δ aly2 rod1Δ rog3*Δ cells, 18%; *rod1Δ rog3Δ csr2Δ ecm21*Δ cells, 69%; and *aly1Δ aly2 csr2Δ ecm21*Δ cells, 70%. Thus, the joint actions of Aly1, Aly2, Rod1, and Rog3 are specifically and primarily responsible for the α-arrestin-dependent internalization of CDT-1 and CDT-2.

Because industrial-scale fermentations are typically carried out anaerobically, we also examined the localization of CDT-1 and CDT-2 under anoxic conditions (as described in Materials and Methods). The distribution of both CDT-1 and CDT-2 observed in WT, the *9arr*Δ strain, and *4arr*Δ cells grown in cellobiose medium under anaerobic conditions was quite similar to that observed under aerobic conditions (see Fig. S3 in the supplemental material).

### Anaerobic growth and fermentation of cellobiose by CDT-2-expressing cells is improved when the relevant α-arrestins are absent.

**FIG 3:**
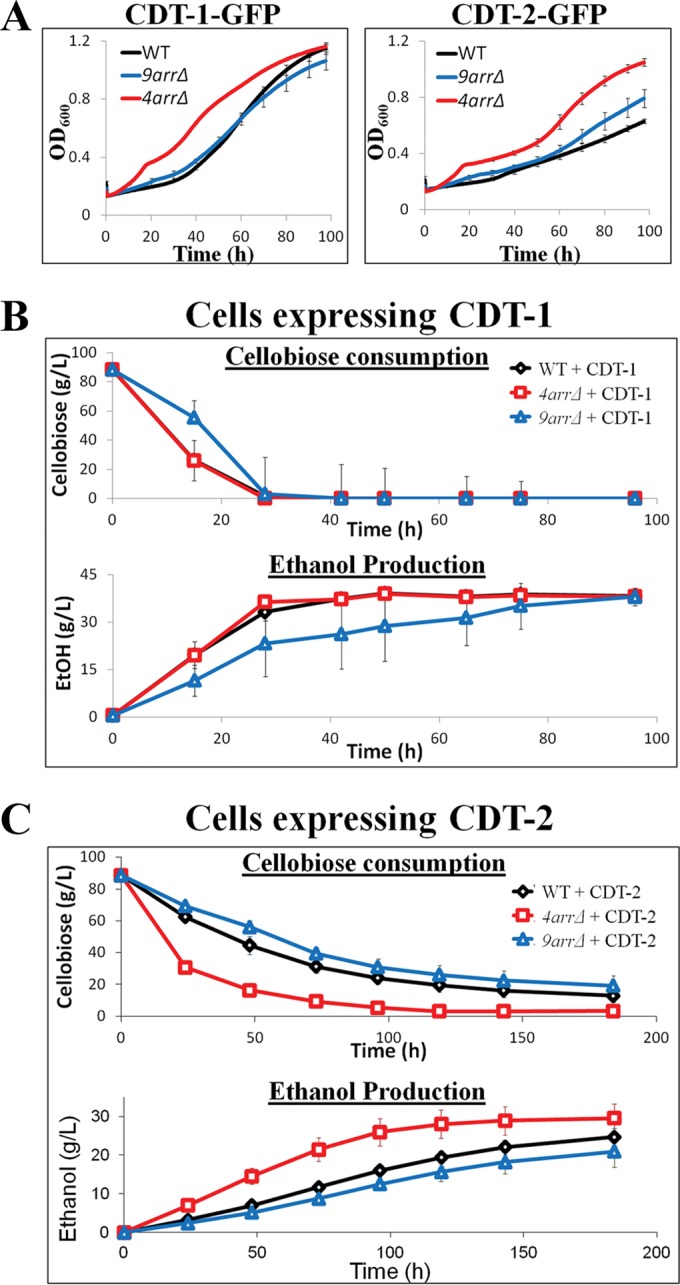
Increased cell surface levels of CDT-1 and CDT-2 in *4arr*Δ cells lead to increased anaerobic growth and increased cellobiose fermentation for CDT-2-expressing cells. (A) Anaerobic growth curves showing the rate of growth for cells expressing CDT-1 or CDT-2 in BY4741 (WT) and the derivative *9arr*Δ and *4arr*Δ strains. (B and C) Cellobiose consumption and ethanol production were measured for WT, *9arr*Δ, and *4arr*Δ strains expressing CDT-1 (B) and CDT-2 (C). The intracellular β-glucosidase (GH1-1) was always coexpressed in the experiments whose results are shown in panels A, B, and C.

We found that the cellobiose transporters were localized almost exclusively at the PM in both the *9arr*Δ and *4arr*Δ cells. This observation raised the possibility that a higher steady-state level of these transporters might increase the transmembrane flux of cellobiose and, if the rate of entry of the disaccharide is a factor limiting its utilization, might result in more efficient growth and fermentation of cellobiose to ethanol. Because it has been observed before that the *9arr*Δ strain is somewhat compromised for growth (25), presumably because of the cumulative deleterious effects arising from the simultaneous absence of nine α-arrestins, we were most interested in the *4arr*Δ cells. Indeed, when growth assays were performed in cellobiose medium under anaerobic conditions, *4arr*Δ cells expressing either CDT-1 or CDT-2 exhibited both a faster doubling time and a higher final growth yield than those of otherwise isogenic WT cells (or the *9arr*Δ strain) (Fig. 3A).

We also examined whether the elevated PM localization of the cellobiose transporters would enhance cellobiose utilization and, concomitantly, the efficiency of ethanol production. For *4arr*Δ cells expressing CDT-1, the rate and extent of cellobiose consumption and the rate and extent of ethanol production were not detectably different from those of otherwise isogenic WT cells expressing CDT-1 (Fig. 3B). In marked contrast, we observed that *4arr*Δ cells expressing CDT-2 exhibited a rate and extent of cellobiose consumption and a rate and extent of ethanol production that were reproducibly better than those of otherwise isogenic WT cells (or the *9arr*Δ strain) expressing CDT-2 (Fig. 3C). One consideration that may explain why an effect of removing the relevant α-arrestins was detectable for CDT-2-expressing cells, but not for CDT-1-expressing cells, is that the reported maximum rate of metabolism (*V*_max_) for cellobiose uptake catalyzed by CDT-1 is more than 2-fold higher than that mediated by CDT-2 (12). Thus, removal of the relevant α-arrestins may result in a greater incremental increase in cellobiose entry in cells expressing CDT-2 than in cells expressing CDT-1.

### C-terminal lysine residues of CDT-2 are important for its internalization.

CDT-1, an ATP-driven proton symporter, supports a higher rate of cellobiose entry than CDT-2, which mediates cellobiose entry by facilitated diffusion (12, 42). Because cellobiose uptake by CDT-1 is coupled to ATP consumption, less of the cellobiose can, in principle, be converted to ethanol. Thus, CDT-2 is a potentially more attractive alternative for industrial-scale conversion of cellobiose to ethanol, despite the fact that it is less efficient in supporting cellobiose fermentation (43). However, as we have demonstrated here, removal of the cognate α-arrestins that mediate the ubiquitinylation-dependent internalization of CDT-2 provided significant improvement in cellobiose utilization and ethanol production. Given those findings, we reasoned that an alternative strategy to enhance the amount of CDT-2 in the PM and thereby increase cellobiose entry would be to identify and eliminate the Lys residues that are the targets of its α-arrestin-dependent modification (as long as the residues substituted for Lys did not compromise the folding, trafficking, and/or transport functions of CDT-2).

**FIG 4:**
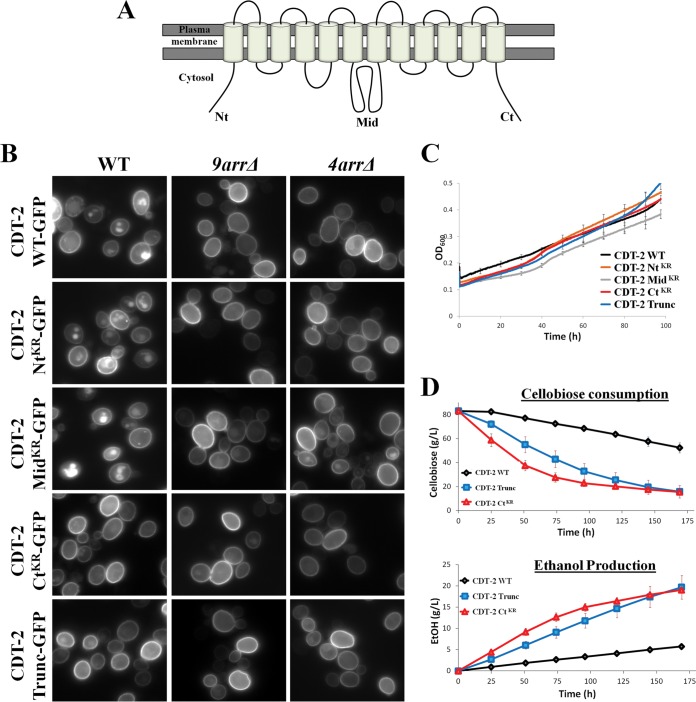
C-terminal mutants of CDT-2 are deficient in endocytosis and show increased cellobiose fermentation. (A) Schematic representation of the topology of CDT-2 on PM. (B) Representative images of Lys-to-Arg mutants of CDT-2 (Nt, N-terminal; Mid, large loop interconnecting the 6th and 7th transmembrane regions; Ct, C-terminal; Trunc, truncation) expressed in WT, *9arr*Δ, and *4arr*Δ cells. It should be noted that the CDT-2 constructs are expressed under the *CCW12* promoter. (C) Anaerobic growth curves of CDT-2 and the derivative mutants expressed in WT cells. (D) Cellobiose consumption and ethanol production for CDT-2 and its mutants expressed in WT cells.

There is no crystal structure available for the CDT-2 transporter. Therefore, we used the I-TASSER Protein Structure Prediction server (44) to model this transporter against crystal structures of homologous members of the SP subgroup of the major facilitator superfamily (see Fig. S4 in the supplemental material). Rather than bias the modeling to conform to any specific known structures, we allowed the I-TASSER algorithm to find the closest match and build the homology model. Based on both hydropathy plots (45) and the homology model, CDT-2 contains 12 transmembrane helices organized into two domains with N- and C-terminal extensions that project into the cytosol (Fig. 4A; see Fig. S4). These cytosolic “tails,” along with the five interconnecting loops that face the cytosol, contain multiple Lys residues, any or all of which might serve as sites for the covalent attachment of ubiquitin mediated by the Aly1-, Aly2-, Rod1-, and Rog3-dependent recruitment of the E3 Rsp5. To determine whether any of the three most prominent cytosolic segments of CDT-2 (its N-terminal extension, its C-terminal extension, and the large, predicted interdomain loop between transmembrane helices 6 and 7) represent sites that contribute to its internalization, all of the Lys residues in each of these regions were mutated to Arg, and these CDT-2 mutants are here called the Nt^KR^ mutant, the Ct^KR^ mutant, and the Mid^KR^ mutant. Strikingly, the Ct^KR^ mutant displayed the enhanced PM localization and markedly reduced vacuolar localization also observed in *9arr*Δ and *4arr*Δ cells, whereas the Nt^KR^ and Mid^KR^ mutants exhibited internalization very similar to that seen in WT cells (Fig. 4B). For 200 total cells in random fields of each culture expressing WT CDT-2–GFP or the indicated CDT-2–GFP mutants, we observed the following percentages of cells with a robust vacuolar signal: WT CDT-2–GFP in WT cells, 83%; WT CDT-2–GFP in *9arr*Δ cells, 11%; WT CDT-2–GFP in *4arr*Δ cells, 19%; CDT-2 Nt^KR^–GFP in WT cells, 80%; CDT-2 Nt^KR^–GFP in *9arr*Δ cells, 9%; CDT-2 Nt^KR^–GFP in *4arr*Δ cells, 14%; CDT-2 Mid^KR^–GFP in WT cells, 89%; CDT-2 Mid^KR^–GFP in *9arr*Δ cells, 19%; CDT-2 Mid^KR^–GFP in *4arr*Δ cells, 24%; CDT-2 Ct^KR^–GFP in WT cells, 11%; CDT-2 Ct^KR^–GFP in *9arr*Δ cells, 8%; CDT-2 Ct^KR^–GFP in *4arr*Δ cells, 10%. These observations suggested that it is the Lys residues in the C-terminal tail of CDT-2 that must be ubiquitinylated as a prerequisite to its endocytosis. Consistent with that conclusion, a version of CDT-2 with a truncated carboxy terminus (CDT-2^Trunc^; arising from a frameshift resulting in a stop codon that causes premature termination) (see Fig. S5) also exhibited enhanced PM localization and markedly reduced vacuolar localization (Fig. 4B). For 200 total cells in random fields of each culture, we observed the following percentages of cells with a robust vacuolar signal: CDT-2^Trunc^–GFP in WT cells, 25%; CDT-2^Trunc^–GFP in *9arr*Δ cells, 11%; CDT-2^Trunc^–GFP in *4arr*Δ cells, 15%. Although these latter constructs were expressed under the *CCW12_prom_*, instead of the formerly used *PGK1_prom_*, we confirmed that the levels of protein expression driven by either promoter are quite similar (see Fig. S6).

Interestingly, in the context of this work, the truncation mutant (CDT-2^Trunc^) was initially generated in a separate project in which *CDT-2* orthologs in other filamentous fungi were identified that likely had similar transporter activity. The ortholog chosen from Fusarium graminearum, here transporter FG, was subjected to random mutagenesis followed by directed evolution, where the selective pressure was the ability to grow in cellobiose-containing medium. This procedure resulted in the isolation of an FG mutant with a single base deletion (A1547) resulting in a frameshift after residue 515, thereby changing the C-terminal sequence to ^516^RLKKRPStop and causing premature termination of this otherwise 544-residue protein (see Fig. S5 in the supplemental material) (unpublished results). The C-terminal region of FG has high sequence identity to CDT-2 (see Fig. S5). Hence, the CDT-2^Trunc^ mutant was created using the same frameshift mutation to assess whether this alteration would increase its proficiency in cellobiose uptake. In light of our current findings, the apparent reason that FG^Trunc^ and CDT-2^Trunc^ are able to support improved cellobiose utilization is that both are able to escape from their α-arrestin-dependent and Rsp5-mediated ubiquitinylation, just like the CDT-2 Ct^KR^ mutant. In this regard, it is noteworthy that, compared to WT CDT-2, CDT-2^Trunc^ lacks only a single Lys residue (K522), and, similarly, compared to WT FG, FG^Trunc^ lacks only two Lys residues (K528 and K543), suggesting that it is the most C-terminally situated Lys residues that are the primary sites for Rsp5-mediated ubiquitinylation of these two proteins.

### Anaerobic fermentation of cellobiose is improved when CDT-2 cannot undergo α-arrestin-dependent internalization.

Given that both the CDT-2 Ct^KR^ and CDT-2^Trunc^ mutants increased PM localization and decreased internalization of this transporter, and are apparently “immune” to α-arrestin-dependent endocytosis, we reasoned that they should support more efficient cellobiose consumption and ethanol production even in WT cells. Indeed, as predicted, even though the overall growth rate was not significantly affected (Fig, 4C), WT cells expressing either CDT-2 Ct^KR^ or CDT-2^Trunc^ displayed markedly improved cellobiose consumption and increased ethanol production compared to the same cells expressing wild-type CDT-2 (Fig. 4D).

### CDT-2 is internalized in an α-arrestin-dependent manner in response to xylan.

In N. crassa, CDT-2, and not CDT-1, permits utilization of xylan (13). If, as we observed for cellobiose, substrate transport serves as a trigger for transporter endocytosis, we then reasoned that for yeast ectopically expressing CDT-1 and CDT-2, the presence of xylan should promote internalization of the latter but not the former. In this regard, and in contrast to the experiments carried out with cellobiose, which were conducted in minimal medium, the experiments performed with xylan were conducted in rich medium (YP), which was necessary to provide sufficient alternative carbon sources for cell survival (because these S. cerevisiae cells do not possess the enzymatic machinery for subsequent xylan utilization).

**FIG 5:**
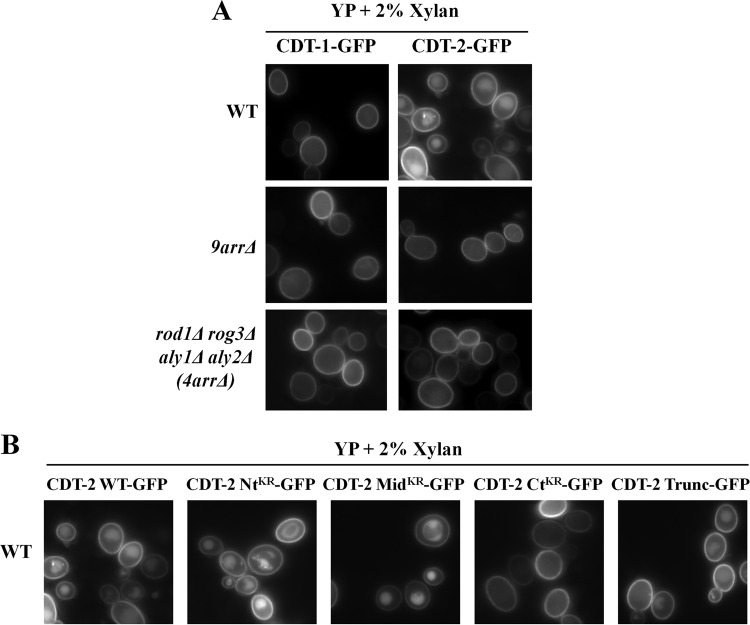
CDT-2, not CDT-1, undergoes α-arrestin-dependent endocytosis in response to xylan. (A) To ensure cell survival of BY4741 and its derivative strains (which do not express the requisite enzymes for xylan metabolism) in the presence of xylan as the carbon source, cultures were grown in rich medium (YP). Representative images were observed for cells expressing CDT-1 or CDT-2. (B) The same experiment as that described for panel A, except that CDT-2 and its derivative mutants were expressed in WT cells.

In agreement with the conclusion that the presence of a transport substrate promotes transporter endocytosis, we found that internalization of CDT-2, but not CDT-1, was stimulated in xylan-containing medium (Fig. 5A). For 200 total cells in random fields of each culture propagated in yeast extract-peptone-dextrose (YPD) medium containing xylan, we observed the following percentages of cells with a robust vacuolar signal: WT CDT-1–GFP in WT cells, 4%; WT CDT-1–GFP in *9arr*Δ cells, 3%; WT CDT-1–GFP in *4arr*Δ cells, 3%; WT CDT-2–GFP in WT cells, 84%; WT CDT-2–GFP in *9arr*Δ cells, 9%; WT CDT-2–GFP in *4arr*Δ cells, 11%. Thus, internalization of CDT-2, but not CDT-1, was stimulated in xylan-containing medium, and, as for cellobiose, CDT-2 internalization in response to xylan was eliminated in both the *9arr*Δ strain and the *4arr*Δ cells. Likewise, compared to wild-type CDT-2, the CDT-2 Ct^KR^ mutant abrogated xylan-stimulated endocytosis almost completely, and the CDT-2^Trunc^ mutant significantly reduced internalization (Fig. 5B). For 200 total cells in random fields of each culture propagated in YPD medium containing xylan, we observed the following percentages of cells with a robust vacuolar signal: WT cells expressing WT CDT-2–GFP, 88%; WT cells expressing CDT-2 Nt^KR^–GFP, 81%; WT cells expressing CDT-2 Mid^KR^–GFP, 89%; WT cells expressing CDT-2 Ct^KR^–GFP, 6%; WT cells expressing CDT-2^Trunc^–GFP, 21%.

## DISCUSSION

CDT-1 and CDT-2 are cellobiose transporters encoded in the Neurospora crassa genome. When heterologously expressed in S. cerevisiae, along with an intracellular β-glucosidase (GH1-1), this yeast becomes capable of directly transporting and utilizing cellobiose without the need for its prior hydrolysis into glucose (12).

In S. cerevisiae, a 14-member family of endocytic adaptors, the α-arrestins, mediates the Rsp5-dependent ubiquitinylation and subsequent internalization of specific integral membrane proteins in response to specific stimuli. For example, α-arrestin Ldb19 mediates internalization of the methionine transporter Mup1 in response to excess exogenous Met (23), α-arrestin Aly2 promotes internalization of the acidic amino acid transporter Dip5 in the presence of surplus Asp (25, 46, 47), and α-arrestin Art5 triggers internalization of the inositol transporter Itr1 when inositol is supplied (25, 48). Similarly, α-arrestin-dependent endocytosis of the glucose transporters Hxt1 and Hxt3 is stimulated by addition to the medium of 2-deoxy-glucose, a nonmetabolizable glucose analog (20), and the general amino acid permease Gap1 and the arginine-specific permease Can1 are internalized in response to their cognate transport substrates (49).

Here we demonstrated that the ectopically expressed cellobiose transporters CDT-1 and CDT-2 are also subject to transport substrate-induced and α-arrestin-dependent endocytosis. Although it appears to be counterintuitive that the presence of the cognate transport substrate (cellobiose in the case of CDT-1 and cellobiose or xylan in the case of CDT-2) triggers internalization of these transporters, it should be recalled that these molecules evolved in the different PM milieu of another organism. It seems likely, therefore, that, when expressed in yeast, the conformational dynamics required for the transport process occasionally causes partial unfolding that exposes epitopes in the transporters that permit their capture by the S. cerevisiae quality control machinery, which includes surveillance of the status of integral PM proteins by the endogenous yeast α-arrestins.

**FIG 6:**
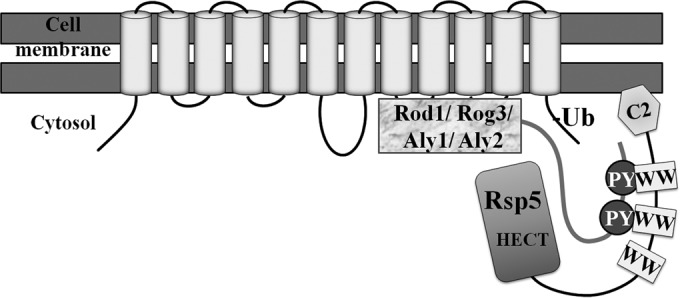
Model for the α-arrestin-mediated endocytosis of the cellobiose transporters. The α-arrestins Rod1, Rog3, Aly1, and Aly2 cooperate to mediate the recruitment of Rsp5 to the ectopically expressed cellobiose transporters. Rsp5 brings about the ubiquitinylation of the transporters, which are subsequently endocytosed.

We found that in S. cerevisiae, CDT-1 and CDT-2 internalization is primarily mediated by just four members of the α-arrestin family: Aly1, Aly2, Rod1, and Rog3. It seems that CDT-1 and CDT-2 must possess sequence and/or structural features that are able to be recognized by each of the these pairs of α-arrestin paralogs, thereby recruiting the ubiquitin ligase Rsp5, which then ubiquitinylates the cellobiose transporters (Fig. 6). Conversely, cells deficient in these four α-arrestins elevate the PM content of these transporters and, as a consequence, allow for increased uptake of cellobiose. Indeed, under anoxic conditions, *4arr*Δ cells expressing either CDT-1 or CDT-2 grow better and, in case of CDT-2, allow for improved conversion of cellobiose to ethanol.

For CDT-2, Lys residues within its C-terminal tail are the most critical for its efficient endocytosis, suggesting that these are the sites where α-arrestin-dependent and Rsp5-mediated ubiquitinylation occurs (Fig. 6). Consistent with α-arrestin-mediated internalization limiting the rate of cellobiose utilization, the CDT-2 Ct^KR^ mutant was localized almost exclusively at the PM and was able to increase cellobiose consumption and ethanol production even in WT cells, just like WT CDT-2 in the α-arrestin-deficient *4arr*Δ cells. These complementary findings indicate that endocytosis of the CDT-2 transporter exerts a rate-limiting effect on cellobiose utilization.

In N. crassa, the CDT-1 and CDT-2 transporters may act as transceptors; they sense and transport the inducer disaccharide cellobiose and induce cellulase production (50). The Neurospora genes *cdt-1* and *cdt-2* are part of the cellulolytic regulon, a group of genes whose expression requires the presence of an inducer molecule (cellulose-derived sugars) and deactivation of carbon catabolite repression (CCR) (51–53). During growth on a preferred carbon source (e.g., glucose or sucrose), the CCR machinery represses the expression of genes in this regulon (54). In both N. crassa and Aspergillus nidulans, the transcription factor CreA/CRE-1 regulates key aspects of CCR. CreA/CRE-1 (54) is a homolog of S. cerevisiae Mig1, a DNA-binding protein that acts in a similar fashion to repress the expression of genes involved in the catabolism of carbon sources other than glucose (55).

In A. nidulans, *creA*, *creB*, and *creC* have been identified as regulators of CCR in genetic screens (56). It is of interest, in light of our results, that *creB* encodes a deubiquitinylating enzyme and *creC* encodes a WD40 motif-containing protein, the hallmark of many F-box-containing proteins. A mutation in *creD* (ANID_04170.1) that suppressed some phenotypic effects of mutations in *creB* and *creC* has been identified (56, 57). Sequence analysis of *creD* showed that it encodes a protein with an N-terminal arrestin N and an arrestin C domain and three PPXY-like motifs and bears significant sequence similarity to the Rod1 and Rog3 α-arrestins of S. cerevisiae (57). Further, CreD interacts with the E3 HulA, the homolog of S. cerevisiae Rsp5. Substrates of CreD in A. nidulans have not been identified, but it is possible that the role of CreD in CCR may involve the endocytosis of transporters of nonpreferred carbon sources, a role analogous to that of Rod1 in S. cerevisiae. We conducted a BLASTP search for homologs of CreD in N. crassa and found that NCU03887 was the closest match, with ∼40% amino acid sequence identity (see Fig. S7 in the supplemental material). To date, NCU03887 function has not been characterized, but, like CreD, it too is potentially a Rod1-like α-arrestin. It is possible that in the context of the posttranslational regulation of CDT-1 and CDT-2, CreD and NCU03887 may regulate the endocytic removal of these cellodextrin transporters from the PM in response to changes in carbon source. These transporters may be necessary to sense and transport cellodextrins at low levels of extracellular cellobiose, but once the full cellulolytic response has been induced and cellulolytic enzymes are actively degrading cellulose to glucose, their presence in the membrane may no longer be necessary.

Taken together, our findings have important implications for the engineering of production strains for the generation of cellulosic biofuels, because they demonstrate that transporter endocytosis can exert a rate-limiting effect on cellobiose utilization.

## Supplementary Material

Supplemental material
