# Supplementary material for: Internalization of Heterologous Sugar Transporters by Endogenous α-Arrestins in the Yeast Saccharomyces cerevisiae
Source: Appl Environ Microbiol. 2016 Nov 21;82(24):7074–85. doi: 10.1128/AEM.02148-16 (PMC5118918; doi:10.1128/AEM.02148-16)
Supplement: Supplemental material [file supp_82_24_7074__index.html]

Supplemental material 

# Internalization of Heterologous Sugar Transporters by Endogenous α-Arrestins in the Yeast Saccharomyces cerevisiae

## Supplemental material

- Supplemental file 1 -

  Amino acid sequence alignment of *N. crassa* CDT-1 and CDT-2 transporters using the PRALINE multiple sequence alignment program because of low sequence identity between CDT-1 and CDT-2 (Fig. S1), visualization of vacuoles (Fig. S2), localization of cells expressing CDT-1 or CDT-2 under normal conditions or after 6 hours of anaerobic growth (Fig. S3), I-TASSER model for CDT-2 (Fig. S4), Clustal Omega alignment of amino acid residues of FG and CDT-2, along with the truncated versions for both (Fig. S5), CDT-2-GFP expression levels (Fig. S6), and Clustal Omega protein sequence alignment (Fig. S7).

  PDF, 4.5M
