# Supplementary material for: Internalization of Heterologous Sugar Transporters by Endogenous α-Arrestins in the Yeast Saccharomyces cerevisiae
Source: Appl Environ Microbiol. 2016 Nov 21;82(24):7074–85. doi: 10.1128/AEM.02148-16 (PMC5118918; doi:10.1128/AEM.02148-16)
Supplement: Supplemental material [file AEM.02148-16_zam999117555so1.pdf]

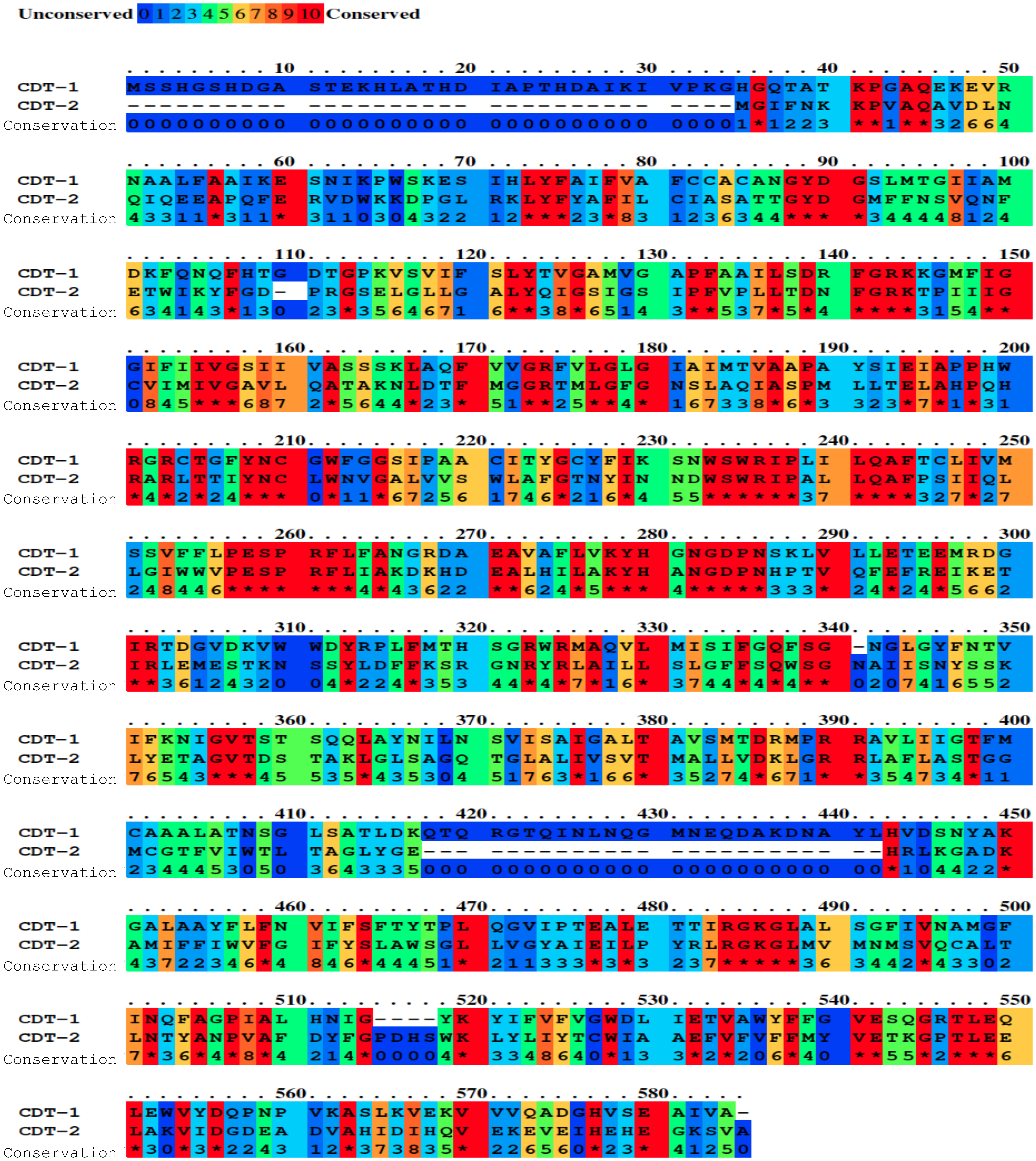

Fig. S1: Amino acid sequence alignment of *N.crassa* CDT-1 and CDT-2 transporters using the PRALINE multiple sequence alignment program because of low sequence identity between CDT-1 and CDT-2 (1). Residues are color-coded based on conservation.

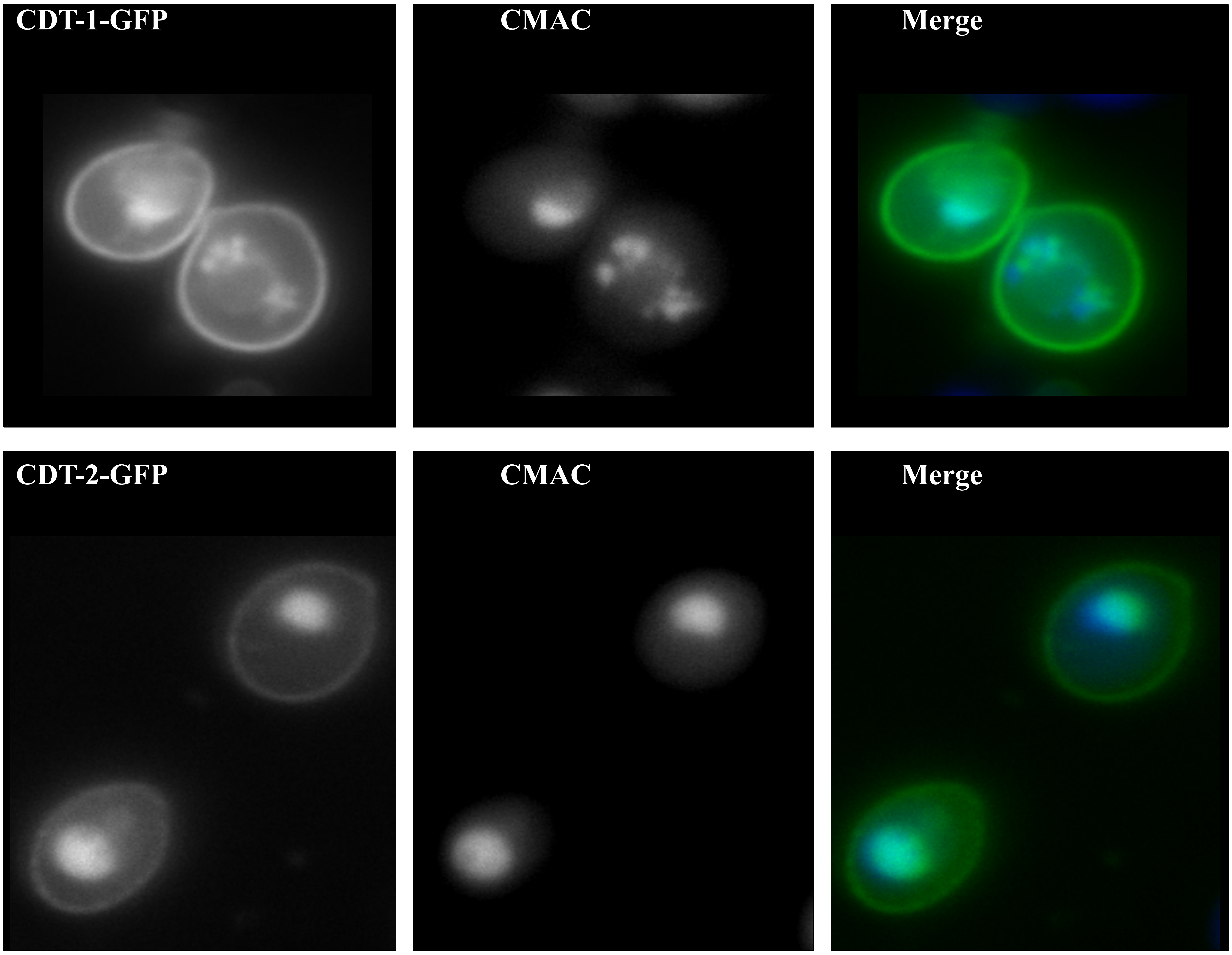

Fig. S2: To visualize vacuoles, WT cells expressing CDT-1-GFP or CDT-GFP were incubated with 250 μM of the vacuolar lumen marker CMAC (Life Technologies, Carlsbad, CA) for 20 min, washed and resuspended prior to imaging.

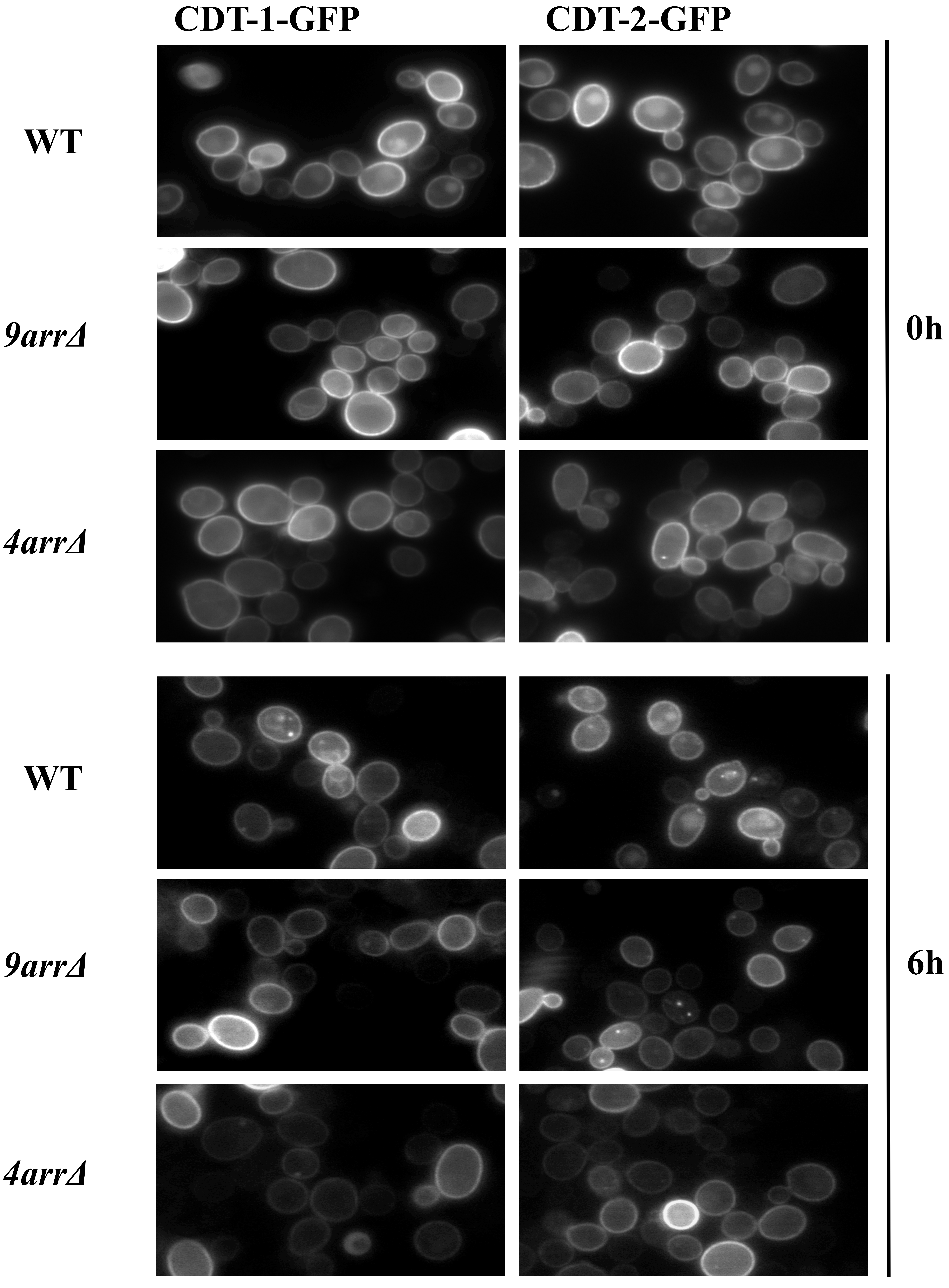

Fig. S3: Localization of WT, *9arrΔ, or 4arrΔ* cells expressing CDT-1 or CDT-2 under normal conditions, or after 6 hours of anaerobic growth.

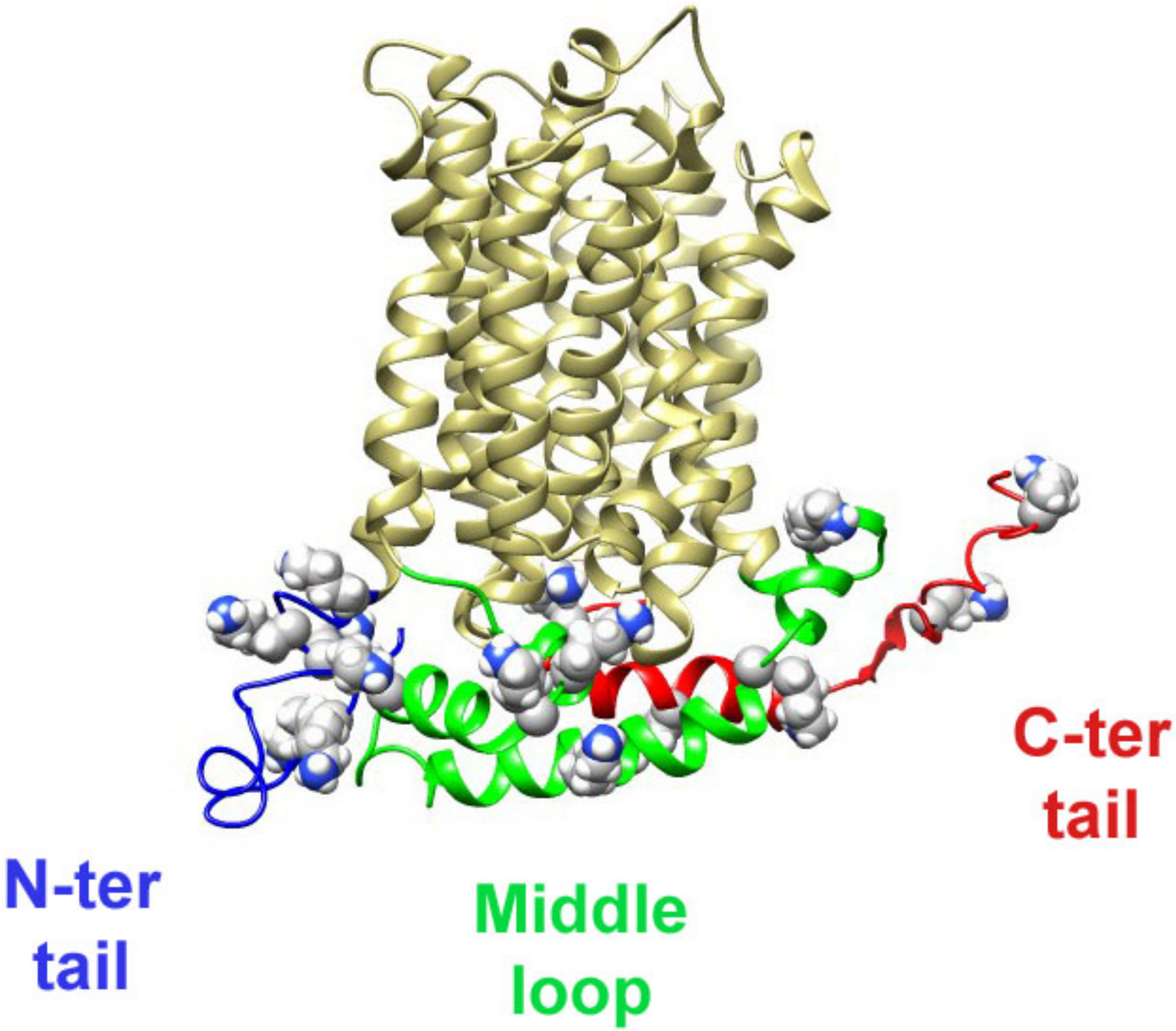

Fig. S4: I-TASSER model for CDT-2 showing Lys residues mutated to Arg in the N-terminal tail (blue), the interdomain middle loop (green), and the C-terminal tail (red).

```
FGTrunc         MGIKEIMGLGADEKTQRLRDEAPKFEKVTWYRDPALRKLTFYACVLCASSMGTGWDGMYM   60
FG              MGIKEIMGLGADEKTQRLRDEAPKFEKVTWYRDPALRKLTFYACVLCASSMGTGWDGMYM   60
CDT-2Trunc      MGIFNKKPVAQAVDLNQIQEEAPQFERVDWKKDPGLRKLYFYAFILCIASATTGYDGMFF   60
CDT-2           MGIFNKKPVAQAVDLNQIQEEAPQFERVDWKKDPGLRKLYFYAFILCIASATTGYDGMFF   60
                *** :   :.   . :::::***:**:* * :**.**** *** :** :*  **:***::

FGTrunc         NNVQNFDSWKNFFDKPEGERLGLLIALYQIGSVASIPLVPLITDRWGRRPSIALGFIIMA   120
FG              NNVQNFDSWKNFFDKPEGERLGLLIALYQIGSVASIPLVPLITDRWGRRPSIALGFIIMA   120
CDT-2Trunc      NSVQNFETWIKYFGDPRGSELGLLGALYQIGSIGSIPFVPLLTDNFGRKTPIIIGCVIMI   120
CDT-2           NSVQNFETWIKYFGDPRGSELGLLGALYQIGSIGSIPFVPLLTDNFGRKTPIIIGCVIMI   120
                *.****::* ::* .*.*..**** *******:.***:***:**.:**:  * :* :**

FGTrunc         IGAGLQAGAPDYATFSGGRVLLGFGNSFAQIASPMLLAELCHPQHRARFTTVYNCLWNLG   180
FG              IGAGLQAGAPDYATFSGGRVLLGFGNSFAQIASPMLLAELCHPQHRARFTTVYNCLWNLG   180
CDT-2Trunc      VGAVLQATAKNLDTFMGGRTMLGFGNSLAQIASPMLLTELAHPQHRARLTTIYNCLWNVG   180
CDT-2           VGAVLQATAKNLDTFMGGRTMLGFGNSLAQIASPMLLTELAHPQHRARLTTIYNCLWNVG   180
                :** *** * :  ** ***.:******:*********:**.*******:**:******:*

FGTrunc         SFLVSWTCFGTSFWGNNWSWRFPAILQGAPGLLQLVVLFWIPESPRFLIAKDRHDEALGI   240
FG              SFLVSWTCFGTSFWGNNWSWRFPAILQGAPGLLQLVVLFWIPESPRFLIAKDRHDEALGI   240
CDT-2Trunc      ALVVSWLAFGTNYINNDWSWRIPALLQAFPSIIQLLGIWWVPESPRFLIAKDKHDEALHI   240
CDT-2           ALVVSWLAFGTNYINNDWSWRIPALLQAFPSIIQLLGIWWVPESPRFLIAKDKHDEALHI   240
                :::*** .***.:  *:****:**:**. *.::**: ::*:***********:***** *

FGTrunc         LAKYHANGDVNHPTVQFEYREIRETIKAEQMADNTSSYIDFFKTKGNRYRLVVLFSLGLF   300
FG              LAKYHANGDVNHPTVQFEYREIRETIKAEQMADNTSSYIDFFKTKGNRYRLVVLFSLGLF   300
CDT-2Trunc      LAKYHANGDPNHPTVQFEFREIKETIRLEMESTKNSSYLDFFKSRGNRYRLAILLSLGFF   300
CDT-2           LAKYHANGDPNHPTVQFEFREIKETIRLEMESTKNSSYLDFFKSRGNRYRLAILLSLGFF   300
                ********* ********:***:***: *  : :.***:****::******.:*:***:*

FGTrunc         SQWSGNGVVSNYSSILYEQAGLESEQDRLIITAGKTILDMIVSIGCALFVDRLNRRFSFL   360
FG              SQWSGNGVVSNYSSILYEQAGLESEQDRLIITAGKTILDMIVSIGCALFVDRLNRRFSFL   360
CDT-2Trunc      SQWSGNAIISNYSSKLYETAGVTDSTAKLGLSAGQTGLALIVSVTMALLVDKLGRRLAFL   360
CDT-2           SQWSGNAIISNYSSKLYETAGVTDSTAKLGLSAGQTGLALIVSVTMALLVDKLGRRLAFL   360
                ******.::***** *** **: ..  :* ::**:* * :***:  **:**:* **::**

FGTrunc         FATGGMFLVLIFWTLTCGLYEQHQAAGANYGMIFLVWMHGVFYSTCWSGLLIGYAVEILP   420
FG              FATGGMFLVLIFWTLTCGLYEQHQAAGANYGMIFLVWMHGVFYSTCWSGLLIGYAVEILP   420
CDT-2Trunc      ASTGGMCGTFVIWTLTAGLYGEHRLKGADKAMIFFIWVFGIFYSLAWSGLLVGYAIEILP   420
CDT-2           ASTGGMCGTFVIWTLTAGLYGEHRLKGADKAMIFFIWVFGIFYSLAWSGLLVGYAIEILP   420
                 :****  .:::****.*** :*:  **: .***::*:.*:*** .*****:***:****

FGTrunc         YSLRAKGLMILNISVQIALLLNNYLNPLAFKAWQAGDDQIFGGNTWRLYLIYTIWVFGEV   480
FG              YSLRAKGLMILNISVQIALLLNNYLNPLAFKAWQAGDDQIFGGNTWRLYLIYTIWVFGEV   480
CDT-2Trunc      YRLRGKGLMVMNMSVQCALTLNTYANPVAFDYFGP-------DHSWKLYLIYTCWIAAEF   473
CDT-2           YRLRGKGLMVMNMSVQCALTLNTYANPVAFDYFGP-------DHSWKLYLIYTCWIAAEF   473
                * **.****::*:*** ** **.* **:**. :          .:*:****** *: .*.

FGTrunc         TFIYFMYVETRGPTLEEVAKVIDGDNAAVGHVTMERLKKRP *                    521
FG              TFIYFMYVETRGPTLEEVAKVIDGDNAAVGHVTMEKVEKEALNEEVDKISSEGQQIHEVA   540
CDT-2Trunc      VFVFFMYVETKGPTLEELAKVIDGDEADVAHIDIHRLKKRP *                    514
CDT-2           VFVFFMYVETKGPTLEELAKVIDGDEADVAHIDIHQVEKEVEIHEHEGKSVA *         525
                .*::******:******:*******:* *.*: :.:::*.

FGTrunc                521
FG              PTKV   544
CDT-2Trunc             514
CDT-2                  525
```

Fig. S5: Clustal Omega (Ver 1.2.1) alignment (2) of amino acid residues of FG and CDT-2, along with the truncated versions for both. FG and CDT-2 share 58.7% amino acid sequence identity.

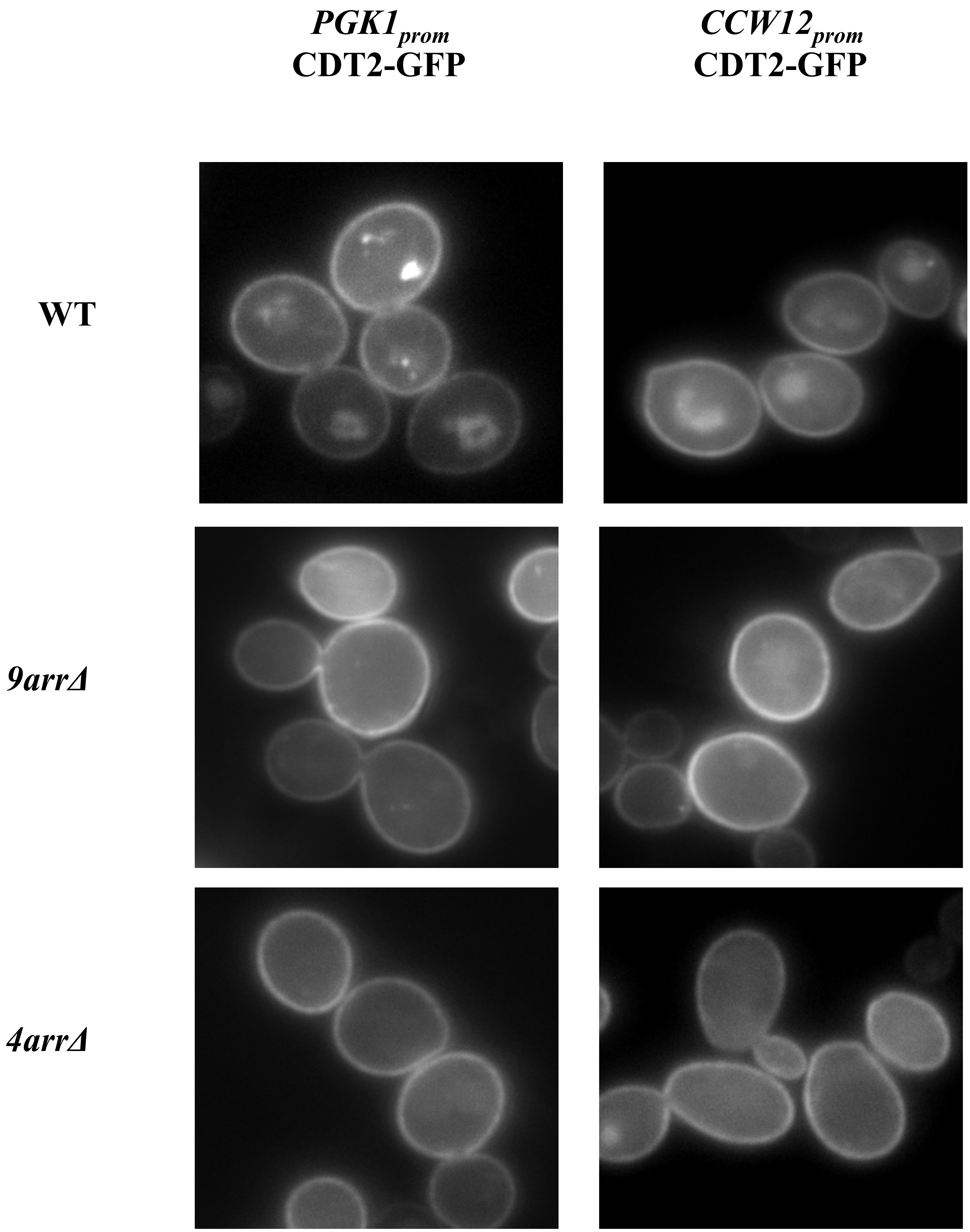

Fig. S6: Representative images showing equivalent levels of CDT-2-GFP expression under the control of either the *PGK1prom* or the *CCW12prom*, as indicated.

```
creD         -MALSFFSGGGSASHAKYFDIRLDEDYIVFRGGEQEAASAHLSGKLVLCVSEPISIKHIR 59

NCU03887     MSFTNFFTSVTGKHAYTYFDIRLESDFIVFRGNEHESAGQLLQGTVVLCLPAPLKIEDVH 60
                 .**:.  .    .******:.*:***** *:*:*.  *.*.:***:  *:.*:.::
creD         LHLTGISRVCWHLPSSSAGGGRKN--WRERVFYEKTWKFRD-A------------GKSKT 104
NCU03887     LRLTGTLHMSWNDPRVTAAGISNHKIDRTSTIYSHRWKPFVGVGAENQSIGSPNGLMSRG 120
             *:***  ::.*. *  :*.*  :.    *  .:*.: **     .             *:
creD         EILPAGNYEYPFDVILEGSMPESVEGLSDTYVTYRFKAEIGR-KYAKDIVVRRPLRIIRT 163
NCU03887     VTLPAGNYEWPFELMLPGDMTESVEGLREASLTYKLKATVARGKLAYDLHAYKRLRIIRT 180
               *******:**:::* *.* ****** :: :**::** :.* * * *: . : ******
creD         LESSALELSHAMSVENIWPNKIEYSISTPTKAVIFGTSIRVDFKLIPLLKGLGIGQIISQ 223
NCU03887     LDPSTLEFLHTMSVENIWPNKIEYSIMIPKKAVVFGSTIPLQTRFTPLLKGLELGEITIR 240
             *: *:**: *:***************  *.***:**::* :: :: ****** :*:*  :
creD         LIETHDLTLNPEDPDAIRNTYKTTRTIINDEHTIDEE--NSLEIIDEAAEGFQFSRTLDL 281
NCU03887     LLEVHEFILQSHSGYGVREHKKEREISVW-KIPIEREKHWQDVIEDTGQEGWVMNTGLDL 299
             *:*.*:: *: ..  .:*:  *   .  :  :  *:.*    .  * * . **: :.  ***
creD         PKTLTRCLQDTDTRGIKVRHKLKFRVQLLNPDGHISELRATLPVSIFISPNLAIDDNNNL 341
NCU03887     PRTLGKCVQDVNAHGIKVRHKLKVVLALHNPDGHISELRATLPVSIFISPNMPLDEEGNL 359
             *:** :*:**.:::*********. : * *********************: :*:: **
creD         VDSSPQTTQRALDDLAQQAPPLYGEHQFDQLYSEVDPSGYRTPGPGSGPGTPFGTLSRNL 401
NCU03887     VRQMPNGTT--PGDVVAAPPPSYDEHTLDQLYEDMEPTGLQTPAGMA---SPLYGHSRAG 414
             * . *: *      *:.   ** * ** :****.:::*:* :**.  :    :*:   **
creD         SAENLASMNAITHTDISASALHHRLVNLDLRGHGRVSASEHDH-LGVPSDNGPPSGSNTH 460
NCU03887     SVENLAALLHMHSTAVPPAALTSRLQNVSLEPSSRSTSWNDGDSETATPNNGPADSV--- 471
             *.****::  :  * :  :**  ** *:.*.  .* :: :. .    .  :*** ..
creD         GSNTHAPGSPELSRRASDEDVHDNIPSGMATPFIPHSAELETLSRVPSYSTAVRSSVRPH 520
NCU03887     --HSSAFPSAPLTRQNSGDNITAEAMSGYHTPEHLDFSGIMELSKVPSYHTAVKTPVRPI 529
               .: *  *  *:*: * :::  :  **  **   . : :  **:**** ***:: ***
creD         ----DSDLPDYQAVVAETVHMSAPQSPQQAHIRGSGTGRGSDSY----FS---------- 562
NCU03887     AYPEGTVLPDYVAATSASG-STTPGSPELGHS---EAGDGASKVEERNQSRPEPPRRKSA 585
                  : **** *..: :   ::* **: .*      :* *:..       *
creD         ---APMDFFHRPAFLHS-----RSHSHSDDERRIR-LTQARGRA  597
NCU03887     RSRLAFTLSHIPHIHHSHSHHGHSHSESEDRRRHSSMMQAN---  626
                  : : * * : **     :***.*:*.**   : **.
```

Fig. S7: Clustal Omega (Ver 1.2.1) protein sequence alignment (2) between *A. nidulans creD* (AN4170) and *N. crassa* NCU03887. These proteins share 42% amino acid sequence identity. The arrestin N and C domains (as predicted by http://pfam.xfam.org/) are highlighted in grey; the PPxY motifs are in red.

Supplemental References

1. **Pirovano, W., Feenstra, K. A., & Heringa, J**. 2008. PRALINETM: a strategy for improved multiple alignment of transmembrane proteins. *Bioinformatics* **24**: 492-497.

2. **Sievers F, Higgins DG**. 2014. Clustal Omega, accurate alignment of very large numbers of sequences. *Methods Mol. Biol.* **1079**: 105-116.
